# The Use of FTIR Spectroscopy as a Tool for the Seasonal Variation Analysis and for the Quality Control of Polysaccharides from Seaweeds

**DOI:** 10.3390/md21090482

**Published:** 2023-09-01

**Authors:** Laurent Vandanjon, Anne-Sophie Burlot, Elando Fréda Zamanileha, Philippe Douzenel, Pierre Hervé Ravelonandro, Nathalie Bourgougnon, Gilles Bedoux

**Affiliations:** 1 Laboratory of Marine Biotechnology and Chemistry (LBCM), University Bretagne Sud (UBS), EMR CNRS 6076, IUEM, Campus Tohannic, 56000 Vannes, France; anne-sophie.burlot@univ-ubs.fr (A.-S.B.); elando.zamanileha@outlook.com (E.F.Z.); nathalie.bourgougnon@univ-ubs.fr (N.B.); gilles.bedoux@univ-ubs.fr (G.B.); 2 Research Unit in Process and Environmental Engineering (URGPGE), Faculty of Sciences, PEI, University of Antananarivo, Antananarivo 101, Madagascar; phravelona@yahoo.com; 3 SVT Department, Faculty of Sciences, UBS, Campus Tohannic, 56000 Vannes, France; philippe.douzenel@univ-ubs.fr

**Keywords:** FTIR spectroscopy, *Eucheuma denticulatum*, *Solieria chordalis*, *Sargassum muticum*, carrageenans, fucoidans, alginates, seasonal variation

## Abstract

Macroalgae are a potentially novel source of nutrition and biologically active molecules. Proliferative species such as *Eucheuma denticulatum*, *Solieria chordalis* (red algae) and *Sargassum muticum* (brown alga) constitute a huge biomass that can be exploited. In this study, we focus on the extraction of polysaccharides from these three macroalgae species and the characterization of cell wall polysaccharides such as carrageenans, fucoidans and alginates by Fourier Transform Infrared spectroscopy with Attenuated Reflectance Module (FTIR-ATR). The comparison of purified extracts with commercial solutions of fucoidans, alginates or carrageenans shows a strong similarity between the spectra. It demonstrates that the methods of extraction that have been used are also suitable purifying technics. Moreover, it validates infrared spectroscopy as a quick, simple and non-destructive method for the accurate analysis of polysaccharides. The FTIR technique applied to samples collected at different periods of the year allowed us to highlight differences in the composition of fucoidans, alginates and carrageenans. Different classes corresponding to the season can be distinguished by statistical multidimensionnal analysis (Principal Component Analysis) showing that the structure of algal polysaccharides, related to bioactivity, depends on the period of harvest. FTIR results showed that *S. chordalis* and *E. denticulatum* possess a dominant type of carrageenan called iota-carrageenan. This type of carrageenan is in the majority when the alga is at maturity in its development cycle. During its growth phase, iota-carrageenan precursors can be detected by FTIR spectra, enabling a better control of the extraction and an application of these compounds in various economic sectors. When the alga *E. denticulatum* is in its juvenile stage, we found traces of kappa-carrageenan and nu-carrageenan polysaccharides in some extracts.

## 1. Introduction

Seaweed is a marine resource used in a wide range of economic sectors, including food, feed, health, agriculture and cosmetics. To meet the demands of these markets, 35.1 million tons of seaweed were cultivated and harvested worldwide in 2019 [1]. Global trade in seaweed and seaweed products amounted to USD 5.6 billion [1]. Sourcing seaweed is a crucial step for any company looking to process it. Seaweed can be cultivated and imported from various countries (e.g., China, Indonesia and the Philippines) [2]. Today, the world’s leading use of macroalgae is for the production by aquaculture of carrageenans and cell wall polysaccharides for the phycocolloid sector [3]. The main sources of carrageenan are some Gigartinales such as *Kappaphycus* sp., followed by *Eucheuma denticulatum*, *Sarcothalia radula* (formerly *Gigartina radula)*, *Sarcopeltis skottsbergii* (formerly *Gigartina skottsbergii)* and, finally, *Chondrus crispus* [4]. Carrageenans are a family of linear sulfated galactans, mainly derived from red algae of the order Gigartinaceae, and are widely used in the food, cosmetics and pharmaceutical industries for their gelling, thickening and stabilizing properties as well as for their biological activities [5]. They are classified according to the presence of the 3,6-anhydro bridge on the four-membered galactose residue and the position and number of sulfate groups.

These polysaccharides can also be traditionally identified as kappa (κ-; G4S-DA) (i.e., Carrageenose 4’-sulfate), iota (ι-; G4S-DA2S) (i.e., Carrageenose 2,4’-disulfate) and lambda (λ-; G2S-D2S,6S) (i.e., Carrageenan 2,6,2’-trisulfate) [6] and the precursors of κ and ι-, respectively, named mu (μ-) and nu (ν-) feature C_6_ sulfate groups, coded D6S (i.e., 4-Linked a-d-galactopyranose 6-sulphate) upon alkaline treatment, whereby this group is converted into DA units (4-Linked 3,6-anhydro-a-d-galactopyranose) [5,6,7].

Several studies show the identification of Rhodophyta red algal carrageenan, mostly carried out by FTIR and NMR [5,7,8,9,10,11]. Since the evolution of studies on algae in recent years, biomaterials based on carrageenans extracted from red algae (Rhodophyta) have been considered due to their versatile qualities, including biodegradability, biocompatibility and non-toxicity, as well as their bioactive attributes, such as antiviral, antibacterial, antihyperlipidemic, anticoagulant, antioxidant, antitumor and immunomodulatory properties [12].

In Madagascar, *Eucheuma denticulatum* (Burman) Collins and Hervey (Rhodophyta, Solieriaceae), known as ‘spinosum’, is widespread along 300 km of coastline between Tulear and Morondava. *Eucheuma denticulatum,* the main source of iota-carrageenan, has been cultivated for 50 years. Its rheological properties were superior to industrial standards, with a highly elastic gel, making it suitable for commercial applications [13]. This species is currently being cultivated and is slowly spreading along the east coast of Madagascar.

In brown algae, two forms of polysaccharides are found simultaneously: alginates and fucose-rich sulfated polysaccharides (fucans and fucoidans). Alginates (or alginic acids) are linear polymers comprising a β-l-guluronic acid unit (GG unit), an α-d-mannuronic acid unit (MM unit) and mixing units (MG unit) linked by one to four bonds [14]. Sulfated fucose-rich polysaccharides are polymers consisting mainly of sulfated fucose (l-conformation) or other sulfated monosaccharides [15]. Brown algal fucoidans have biological activities such as antiviral [16], antioxidant [17], antiproliferative (antitumor) [18], anticomplementary (inhibition of red blood cell lysis) [19], anti-inflammatory [20], antithrombotic [21] and anticoagulant [22]. Balboa et al. [23] suggest that all this variety of activities is linked to the structure of fucoidans, which is highly heterogeneous from one species to another. Fucoidans are also popular in Japan for their health benefits and are consumed as a dietary supplement. Some studies have shown that these compounds enhance the immune system by activating lymphocytes and macrophages [24], improve cell communication and reduce cancer development [25]. Fucoidans have many other uses such as promoting cell regeneration (mobilization of the stem cells) [26] and reducing immunoglobulin E levels involved in allergy phenomena [27].

In Brittany (France), *Solieria chordalis* (C. Agardh) J. Agardh (Rhodophyta, Gigartinales), the cause of major strandings, is periodically found on beaches. Harvested and processed by a local company, extracts from this seaweed are incorporated into products for animal and plant nutrition and health [28]. *Sargassum muticum* (Yendo) Fensholt 1955 (Ochrophyta, Fucales) also found in Brittany, was introduced from Japan [29]. These invasive brown macroalgae form golden tides [30] and have been studied for their active compounds such as polyphenols and for their phycocolloids [31]. For example, *Sargassum* alginates are used for alginate production, mainly in India in the textile industry [32], although these alginates have a low viscosity. On the other hand, in the cold waters of the Atlantic Ocean, other algae with better alginate quality, such as *Laminaria,* are preferred to *Sargassum* for alginate production. According to Gorham and Lewey (1984), fucoidans account for 8% of the dry matter in *S. muticum* [33].

Infrared (IR) spectroscopy is the most useful technique for the rapid and environmentally friendly analysis of biomass. This spectroscopic technique is a fast, accurate and non-destructive tool for on-site detection of certain molecules. There are three main applications: identification, quality control and structural elucidation. The IR method has been used in the agricultural and industrial sectors to rapidly identify important characteristics in order to improve nutritive values (crop nitrogen status, for example) [34]. The IR method is also a feed analysis tool that can be used to determine the chemical composition of a range of feeds, including forages, cereals, by-products and silages [35].

With regard to algae, infrared microspectroscopy has been used to identify agar and carrageenans in red algae or alginates in brown algae. Bedoux et al. (2017) [5] used IR to characterize the polysaccharides of *Rhodymenia pseudopalmata*, *Solieria filiformis*, *Gracilaria cornea* (formerly *Hydropuntia cornea)* (Rhodophyta) and *Sargassum fluitans* (Phaeophyceae) as well as *Halymenia floresii* [36]. The equatorial or axial position of sulfate groups in galactose and mannose residues has been determined in the fucoidans of the brown alga *Sargassum stenophyllum* [37]. More recently, linear regression of the height or integrated area under the Amide II band of Diffuse Reflectance Infrared Fourier Transform Spectroscopy (DRIFTS) has been used to predict seaweed proteins with good predictive performance [38].

In the present study, we propose to use IR technology to monitor the polysaccharide variation frequently encountered in phycocolloids and relate it to various parameters such as algal maturity, culture conditions, and species.

## 2. Results

### 2.1. Polysaccharide Extraction Efficiency

#### 2.1.1. *Eucheuma denticulatum* (N. L. Burman) Collins and Hervey, 1917

**Figure 1:**
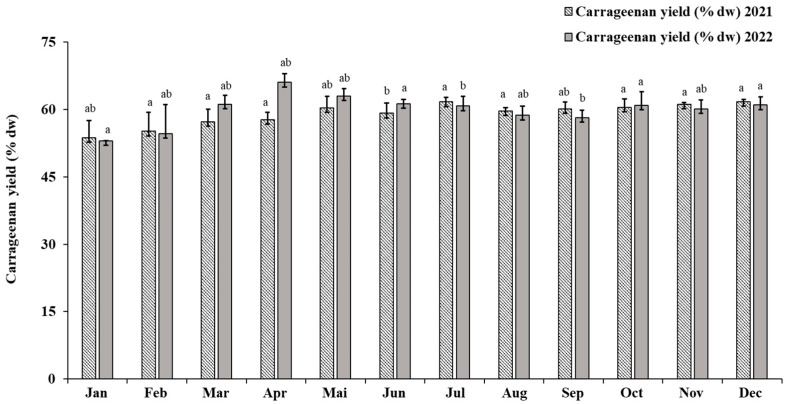
Yields of carrageenan extracted by the conventional hot method using a Thermomix at 80 °C. The difference in bar colors indicates the year of collection, with yield for the year 2021 in striped bars and yield for the year 2022 in gray bars. Significant differences are expressed by letters other than a and b, with *p* < 0.05, sample size n = 3.

Polysaccharides from *E. denticulatum* were extracted in distilled water at 80 °C and precipitated with two volumes of 99% pure ethanol. In general, the extractions gave carrageenan yields in excess of 50% dw of the starting raw material (Figure 1). The yield values obtained vary according to year and collection season. In 2021, the maximum yield is 61.73% dw (corresponding to December) and the minimum yield is 53.74% dw. Whereas for the year 2022, the maximum value reaches 66.03% dw (April) and the minimum is 53.06% dw (January). Similarly, the results of the statistical analysis are significant in terms of yield (*p* < 0.05), with little difference between the carrageenans yields obtained. This difference varies from season to season, given that the abiotic parameters of the algae vary with the growth stage of the species, and its characteristics also change.

#### 2.1.2. *Solieria chordalis* (C. Agardh) J. Agardh

**Figure 2:**
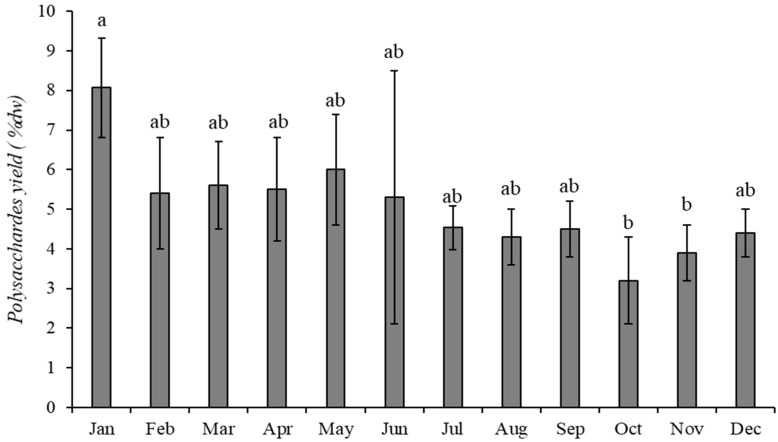
Polysaccharide yield from *Solieria chordalis* for the year 2015. Values with different letters are significantly different (*p* ≤ 0.05; n = 3).

Polysaccharides extraction yields from *Solieria chordalis* are shown in Figure 2. A slight variation in extraction yield is noticeable. Indeed, yields vary from 3.2 ± 1.1% (October 2015) to 8.1 ± 0.1% (January 2015).

#### 2.1.3. *Sargassum muticum* (Yendo) Fensholt

The biochemical composition of *S. muticum* varies throughout the year. The average values for each family of components are as follows: 26.7% protein, 2.3% uronic acids, 8% sulfate compounds, 23.8% mineral matter, 3.2% phenolic compounds and 36% other compounds including sugars. We also observed that protein content is much higher in September than in the other months. In August, on the other hand, the protein content is much lower, in favor of polysaccharides and other components. The min and max values of different compounds are expressed in % of dry matter in Table 1.

### 2.2. Quality Control of the Extraction by Using FTIR

#### 2.2.1. *Eucheuma denticulatum*

**Figure 3:**
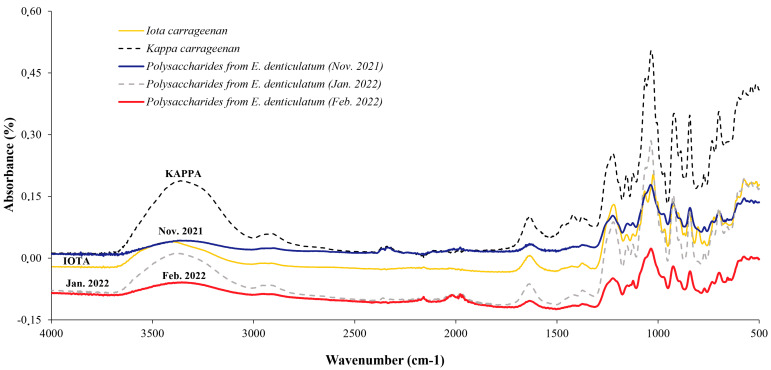
FTIR spectra of iota and kappa commercial carrageenans compared to some examples of carrageenan and polysaccharide extracts from *E. denticulatum* (2021–2022) collected in November 2021, January 2022 and February 2022.

FTIR spectra were obtained directly on *E. denticulatum* polysaccharides from the extraction of carrageenans extracted by the conventional method at 80 °C in a Thermomix during the years 2021–2022. Some spectra are shown in Figure 3 using FTIR spectrometry. This is an application of our methodology described in a paper by Magdugo et al. in 2020 [39]. The spectra showed absorption signals which were compared using commercial carrageenans as a reference (ι- and κ- types). The results show that some samples have traces essentially identical to those of ι-carrageenans, others are identical to κ-carrageenans and some samples show mixtures of ι-, κ- and ν- carrageenans. The method used can therefore provide a rapid qualitative analysis of the type of carrageenan present in samples of whole algae polysaccharides. The band studied for these spectra is the medium-infrared between 4000 and 500 cm^−1^.

#### 2.2.2. *Solieria chordalis*

**Figure 4:**
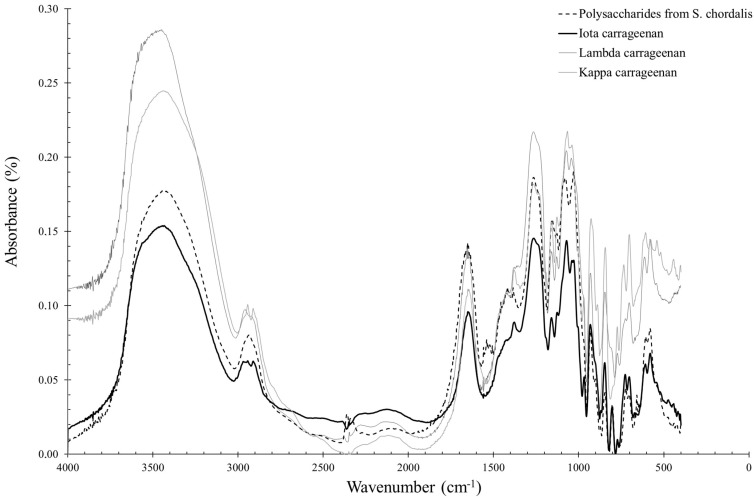
FTIR spectra of iota, lamda and kappa standard carrageenans and polysaccharides from *S. chordalis* harvested in October 2014.

FTIR spectroscopy was used in this study to compare *S. chordalis* polysaccharides collected monthly using a structural and statistical approach. A spectrum of polysaccharides isolated from *S. chordalis* collected in October 2014 is shown in Figure 4. It was compared to the spectra of standard iota-, kappa- and lambda-carrageenans. 

Infrared spectra of polysaccharides from *S. chordalis* samples are superimposable. The characteristic wave numbers of phycocolloids given in the literature have been found. However, the spectra overlap between 580 and 1350 cm^−1^, indicating a seasonal variability of the compounds whose infrared vibrations, i.e., characteristic of chemical bonds, differ.

#### 2.2.3. *Sargassum muticum*

**Figure 5:**
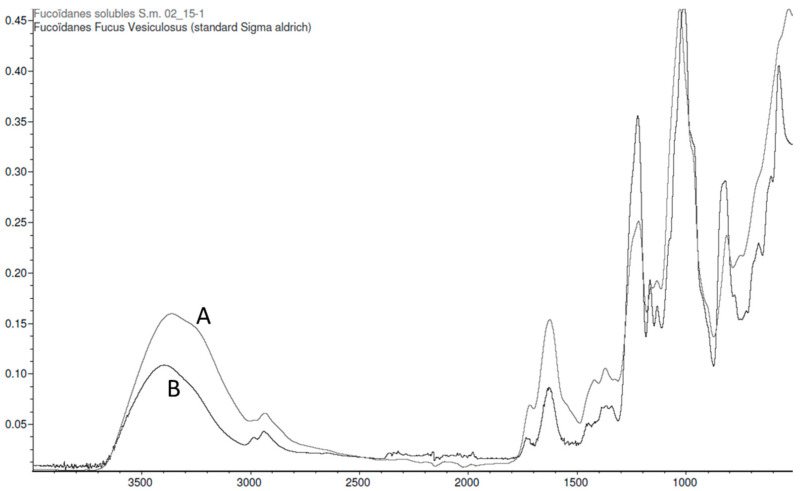
FTIR spectra of a purified extract of soluble fucoidans from *Sargassum muticum* (**A**) and a fucoidan commercial standard (99% purified) from *Fucus vesiculosus* (**B**).

Following the extraction of fucoidans from *Sargassum muticum*, the quality of the extraction can be assessed using purified commercial standards from brown algae (*Fucus vesiculosus*). The comparison of the absorbance spectra (extract and standard in the range 400–4000 cm^−1^) gives a level of similarity of 78.56%. The spectra of fucoidans are presented here (Figure 5) with the main characteristic bands of fucoidans (1220 cm^−1^, S=O; 1020 cm^−1^, O=S=O; 820 cm^−1^, C-O-S).

**Figure 6:**
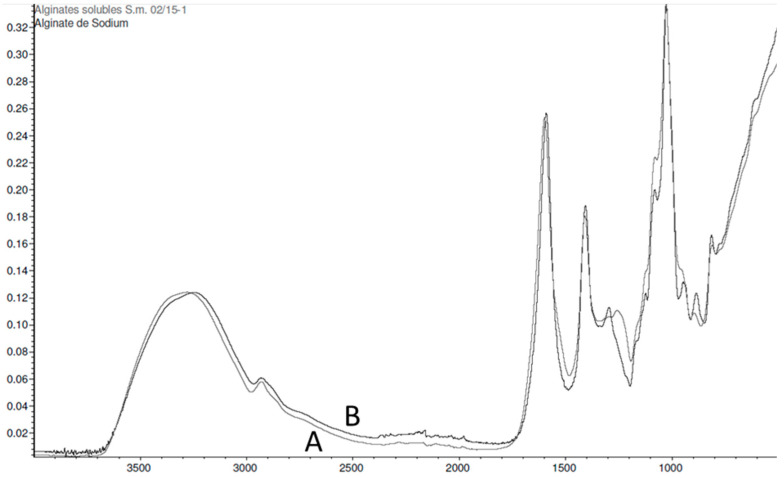
FTIR spectra of a purified soluble alginate extract from *Sargassum muticum* (**A**) and a purified standard commercial alginate (**B**).

The same types of results are obtained with alginates (Figure 6). The comparison of the extracts with a commercial sodium alginate shows that the spectra present a level of similarity of 92.92%. The main characteristic bands of alginates are identified at 1600 cm^−1^, C-C and C=O, 1410 cm^−1^, C-OH, 1026 cm^−1^, O=S=O, 1081 cm^−1^ and 797 cm^−1^ of guluronic acid.

Fucoidans and alginates extracted from *Sargassum muticum* provide accurate absorption bands with the same characteristics as commercial fucoidans and alginates. Thus, a high purity of fucoidan and alginate extracts was demonstrated by FTIR.

### 2.3. Influence of Seasonality on Polysaccharides

#### 2.3.1. *Eucheuma denticulatum*

The analysis of the spectra was referenced with a ι and κ carrageenan standard. The reference band is between 1240 and 800 cm^−1^. According to the literature [40,41], polysaccharide structures can be identified from the following bands: 1240 cm^−1^, S=O of the ester-sulfate is identified by carrageenan ι-, κ- and ν-; 1070 cm^−1^ (shouldered), C-O of 3,6-anhydrogalactose (DA: 4-Linked 3,6-anhydro-α-d-galactopyranose) for ι-, κ-; band 970–975 cm^−1^, Galactose (G/D: 3-Linked β-d-galactopyranose/4-Linked α-d-galactopyranose) of ι-, κ- and ν- (shouldered peaks); 930 cm^−1^ C-O of 3,6-anhydrogalactose (DA: 4-Linked 3,6-anhydro-α-d-galactopyranose) specific for ι- and κ- ; intensity at 905 cm^−1^ (shouldered), C-O-SO_4_ (group ester) on C_2_ of 3,6-anhydrogalactose (DA2S: 4-Linked 3,6-anhydro-α-d-galactopyranose 2-sulphate) specific for ι-carrageenan; 867 cm^−1^ (shouldered), C-O-SO_4_ on C_6_ galactose (G/D6S: 3-Linked β-d-galactopyranose / 4-Linked α-d-galactopyranose 6-sulphate) for ν-carrageenan; 845 cm^−1^ is C-O-SO_4_ on galactose C_4_ (G4S: 3-Linked β-d-galactopyranose 4-sulphate) for ι-, κ- and ν-; band 825–830 cm^−1^, C-O-SO_4_ on galactose C_2_ (G/D2S: 3-Linked β-d-galactopyranose /3-Linked β-d-galactopyranose 2-sulphate) specific for ν-carrageenan ; 815–820 cm^−1^ C-O-SO_4_ on galactose C_6_ (G/D6S: 3-Linked β-d-galactopyranose / 4-Linked α-d-galactopyranose 6-sulphate) for ν-carrageenan and for intensity 805 cm^−1^ C-O-SO_4_ on 3,6-anhydrogalactose C_2_ (DA2S: 4-Linked 3,6-anhydro-α-d-galactopyranose 2-sulphate) specific for ι-carrageenan. Table 2 shows the results of identifying the peak traces to identify the molecular structures of the extracts, and this table already shows the difference of the peaks for each season.

**Figure 7:**
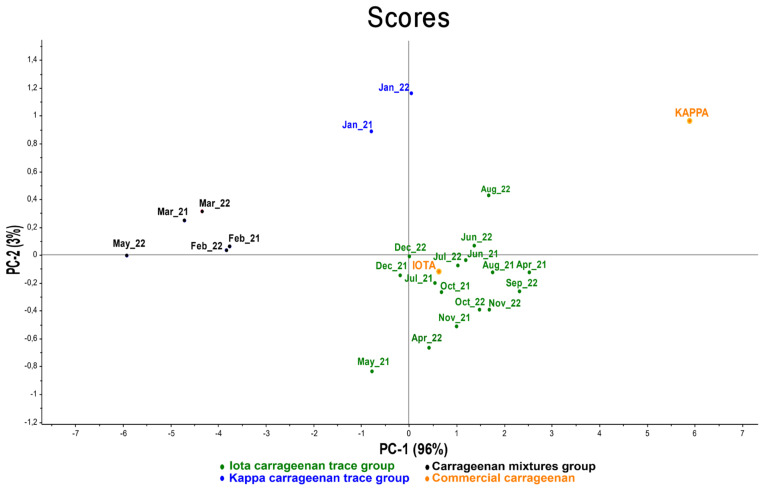
Distribution of *Eucheuma denticulatum* carrageenans by PCA according to the season.

The extracts show high intensity spectral bands at 1232 and 840 cm^−1^, while medium or low intensity bands are located at 1600–1300 cm^−1^ and 840–400 cm^−1^. All spectral bands between 1260 and 1220 cm^−1^ are related to the level of sulfation [42]. All the extracts showed spectral bands at 1240–1218 cm^−1^, corresponding to S=0 of the sulfate ester, up to 1022 cm^−1^, in which the peaks are very intense, due to the elongation of the sulfate group and C-O bonds in the ring groups. The medium and low intensity bands in the 1600–1300 cm^−1^ bands are due to carboxylic acid vibrations. Similarly, the peaks in the 1570–1540 cm^−1^ bands are vibrations of N-H amide bonds [43]. The band at 871 cm^−1^ is characteristic of the presence of sulfate groups on the molecule. Thus, in our extract, the specific peaks of the ν-carrageenan type are few, and only observed in G/D6S* at 871 cm^−1^ but with moderately low intensity. In contrast, traces of ι-, κ- and ν- carrageenan peaks are well highlighted and we find that most of the peaks have ι-type traces. In addition to this identification, we performed a PCA (Principal Component Analysis) study to distinguish polysaccharides (Figure 7). This statistical analysis shows that the majority of the extracts are ι-type carrageenans.

Principal Component Analysis (PCA) is an extremely powerful tool for compressing and synthesizing information, which is especially useful when there is a large amount of quantitative data to process and interpret [44]. Twenty-five samples were analyzed by FTIR, coded by month and year of sampling. We found that almost all samples were correlated with type ι-carrageenan, except January, which was slightly correlated with type κ-carrageenan, and February, March (2021–2022) and May 2022, which were slightly correlated with a type ι-, κ- and ν- carrageenan trace. These mixtures are due to variations in the biochemical composition of the algae, and some elongation of the molecular structures was also observed in some spectra which may be due to extraction.

#### 2.3.2. *Solieria chordalis*

**Figure 8:**
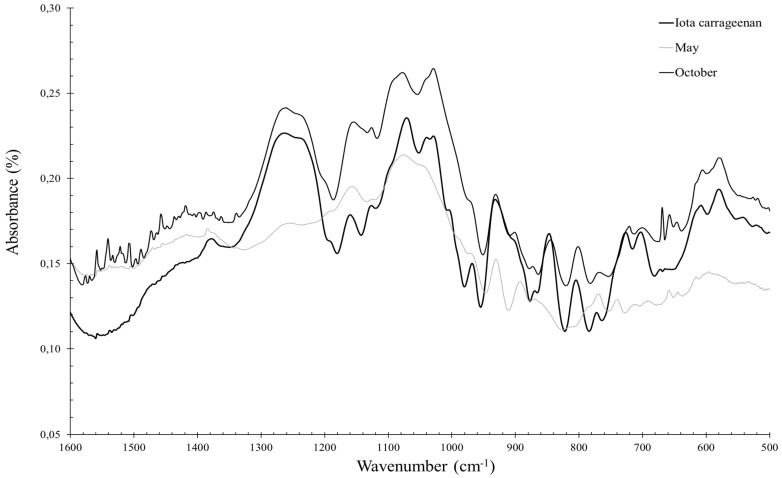
FTIR spectra of iota standard carrageenans and polysaccharides from *S. chordalis* collected in May and October 2014.

The FTIR spectra of polysaccharides extracted from *S. chordalis* samples harvested between September and December are close to the FTIR spectra of standard iota-carrageenan (Figure 8). This type of carrageenan is then dominant at this stage of the seaweed development. In contrast, the FTIR spectra of polysaccharides from *S. chordalis* collected between January and September deviates from that of standard iota-carrageenan. This suggests the presence of carrageenan types other than iota.

**Figure 9:**
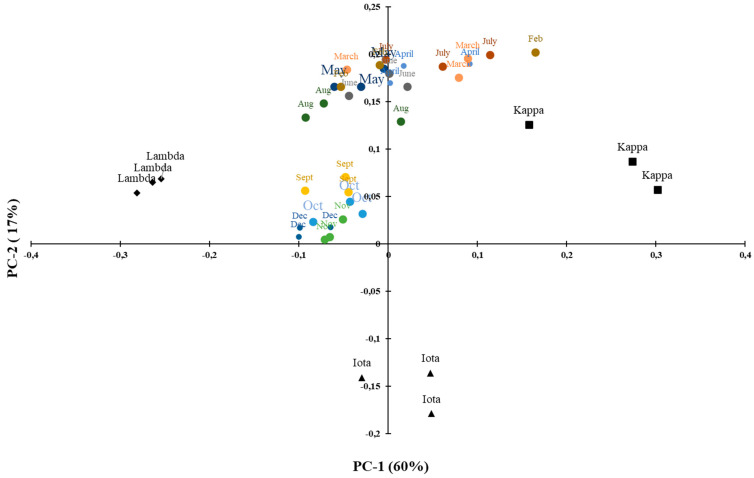
Principal Component Analysis of infrared spectra of *S. chordalis* polysaccharides collected monthly and compared to standard iota, kappa and lambda carrageenans.

A principal component analysis (PCA) was performed to interpret the variability observed on the infrared spectra. Polysaccharides with similar infrared spectra, and therefore similar structures, have been grouped together. PCA was applied after crossing a table containing individuals, corresponding to polysaccharides isolated from *S. chordalis* harvested every month in 2014 and quantitative variables, consisting of spectra obtained by FTIR spectroscopy, over an interval of wavenumber ranging from 580 to 1350 cm^−1^. A significant variability between the spectra explains this choice of analyzed wavenumber interval (Figure 4). A PCA representation of the individuals is obtained and shown in Figure 9.

The selected PCA axes (PC-1 and PC-2) express 77% of the total variability of the infrared spectra of *S.chordalis* polysaccharides obtained at different times of the year. PCA results show two groups of polysaccharides. A first group consists of polysaccharides isolated from samples collected at the end of summer and in autumn (September to December). The other group is more dispersed, gathering polysaccharides from samples of *S. chordalis* collected from February to August. The seasonal variation is clearly noticeable in the characteristics of the polysaccharides. This variation in their structures could be associated with the life cycle of *S. chordalis* (Figure 9). 

#### 2.3.3. *Sargassum muticum*

**Figure 10:**
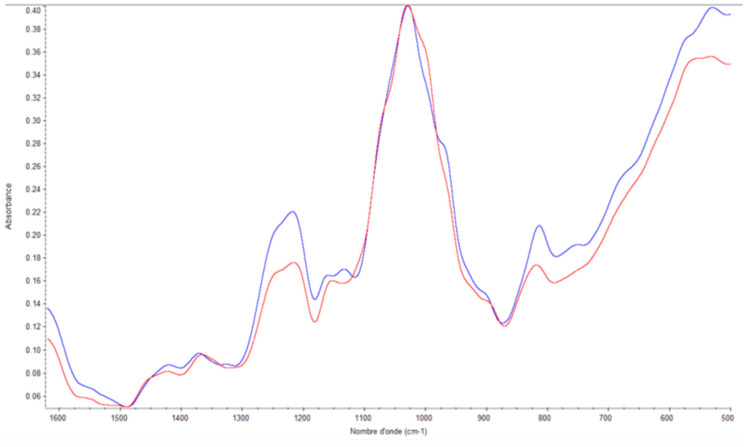
FTIR spectra of soluble *Sargassum muticum* fucoidans collected in February (blue spectrum) and June (red spectrum).

The high level of accuracy of FTIR analyses can be used to monitor seasonal changes in polysaccharide contents of algae. In Figure 10, as an example, the spectra of two samples of soluble fucoidans from *Sargassum muticum* (collected in February and June) are compared in the range 500–1600 cm^−1^.

Although the two spectra are slightly different from each other (depending on the season), they are still close to the fucoidan standard of *Fucus vesiculosus*.

The percentages of similarity of the standard with the samples (triplicates) of soluble fucoidans collected at different months of the year are presented in Table 3.

**Figure 11:**
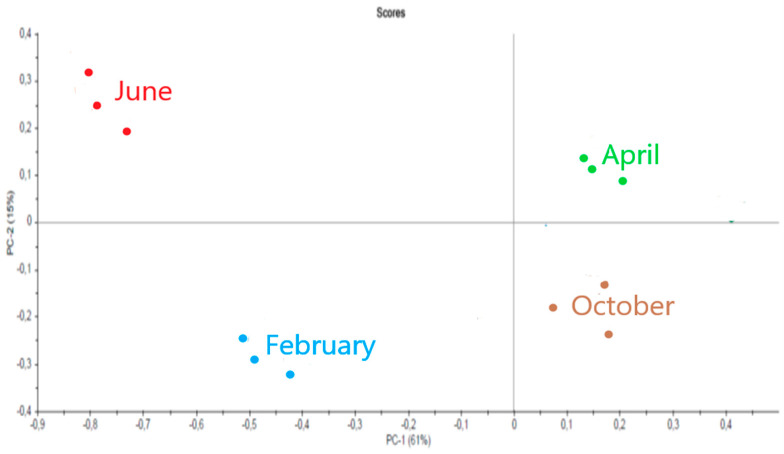
Comparison of fucoidan spectra by PCA (summer, red; spring, green; autumn, brown; winter, blue).

The spectra were then compared to each other using the multidimensional statistical method Principal Component Analysis (PCA) (Figure 11).

**Figure 12:**
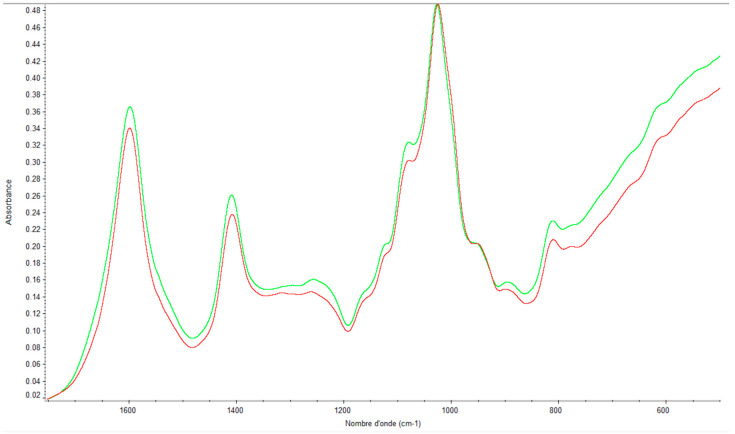
FTIR spectra of soluble *Sargassum muticum* alginates collected in February (green spectrum) and June (red spectrum).

We repeated the same study with alginates and again observed a difference between the spectra depending on the month of collection (i.e, June or February in Figure 12) of *Sargassum muticum*.

**Figure 13:**
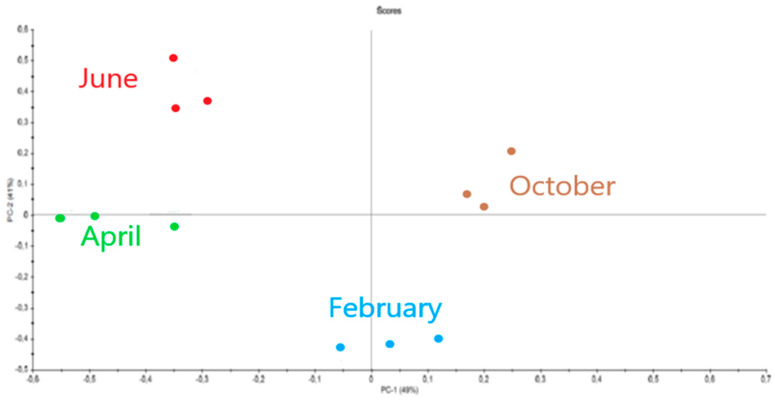
Comparison of alginate spectra by PCA (summer, red; spring, green; autumn, brown; winter, blue).

The PCA comparison of spectra as a function of sampling month is given in Figure 13.

For each sample (measured in triplicate), we notice that the values are grouped together. The spectra of alginates are distinguished into four types corresponding to the four seasons.

## 3. Discussion

### 3.1. Eucheuma denticulatum 

The yields of polysaccharides extracted from *E. denticulatum* varied little during the different seasons, ranging from 53.06% to 66.03%. As the juvenile phase of the algae matures (January–February), the polysaccharide yields are somewhat lower than in the other phases, because the algae store many nutrients for their growth. From this month, the yields are relatively stable, but increase slightly in April and May, when the algae reach maturity (about 90 days) [45]. The mature seaweed is then harvested and the unharvested remains reproduce, multiply again and become more and more thalamus, which is why the yields stabilize from May. The more the *E. denticulatum* is mature, the higher the yield of polysaccharides.

The FTIR spectra of commercial carrageenan and carrageenan from extracts of *E. denticulatum* are compared by peak persistence. The spectra show us the variability of the peaks of each sample according to the seasons, with three groups of spectra found in relation to those of the sample. Two samples are identical in kappa carrageenan correlation in January, when the algae are collected in their juvenile state, and the amount of carrageenan produced in this month is also the lowest compared to other months. Five samples (February, March 2021–2022 and May 2022) contain essentially the same mixtures of carrageenans, with traces of three specific peaks of ι-, κ- and ν-type observed in these samples; therefore, FTIR is not sufficient to identify this structure. During this period, the alga is in transition to maturity and the biochemical composition of the alga also changes at this stage, so we must use another method to properly characterize this mixture. Furthermore, most of the samples are iota-type carrageenans. In most of the spectra, we found a wavenumber of 930 cm^−1^ due to the C-O-C vibration, 1240 cm^−1^ due to the S=O vibration and 840–845 cm^−1^ due to the axial substituent -O-SO_3_ in C_4_ of the β-d-galactopyranosyl unit linked to 3 [46,47,48]. The presence of carboxylic acid vibrations and N-H amide bonds was observed in all spectra, which may be due to protein or amino acid binding in the polysaccharide.

Briefly, it was found that the major sugar units in the polysaccharides of these red seaweed extracts were only galactose and 3,6-anhydrogalactose, implying that the carrageenan molecules are not strongly bound to other types of polymers in the cell wall matrix. In addition to seasonal variations, the structure and carrageenan yield of *E. denticulatum* are also variable, with low yields in summer and higher yields in winter. The carrageenan structure observed in *E. denticulatum* from Madagascar is of the ι-, κ- and ν-type, with most of the structure often consisting of ι-carrageenan.

### 3.2. Solieria chordalis 

In winter and spring (January to June), higher yields of polysaccharides were measured (5.6% to 6.3% of dry weight), compared to those obtained from *S. chordalis* harvested in summer and autumn (3.8% to 4.0% of dry weight). These results (annual average of 5.0 ± 1.2% dried weight) do not match the *S. chordalis* polysaccharide yields measured on samples harvested in Brest in 1984 [49] and 2006 [50]. They are very low in this study. Indeed, Deslandes et al. found a carrageenan yield of 24% of dry weight [49] and Bondu et al. found it to be 14.6% [50], whereas this yield can reach almost 40% of dry weight in some Gigartinales [49,50,51]. It may be associated with the fact that the protocol used in the present study was deliberately designed to be rapid and without a dialysis step. Isolation of polysaccharide took 4 h, whereas the extraction time required is usually more than ten hours or even several days [52,53]. The aim was to coarsely remove pigments, lipids and proteins, and isolate polysaccharides, without necessarily going as far as purifying the carrageenans of *S. chordalis*. Ethanol precipitation concentrates sulfated polysaccharides, such as carrageenans present in *S. chordalis* [54,55,56,57,58]. The seasonal variation in polysaccharide yield supports the idea that parietal synthesis begins in autumn. In addition, at this time of the year, when the alga develops, in particular by multiplying its cells and producing ramules, new walls are synthesized, surrounding each new cell. 

In autumn (October–December), the alga would have reached its stage of maturity, which is confirmed by the identification of iota carrageenans close to this group. Deslandes et al. reported that *S. chordalis* possessed a dominant type of carrageenan called the iota [49]. However, the results of this present study suggest that there are other types of carrageenans and surely hybrids and precursor carrageenans such as ν-carrageenans, or even the more sulfated λ-carrageenans [59,60,61,62]. Hardouin et al. support this hypothesis by reporting that species of the genus Solieriaceae possess mainly ι-carrageenans, but also methylated ι-carrageenans, α-pyruvate carrageenans and ν-type carrageenan precursors [63]. Furthermore, another study confirms this hypothesis of the presence of ν-carrageenans in *S. chordalis* from Brittany [6] or *S. filiformis* [64]. The infrared spectra of these other types of carrageenan are difficult to obtain, as there are no commercial standards unlike iota-, kappa- and lambda-carrageenans. Nevertheless, the methods of extraction and purification of carrageenans from *S. chordalis* used in this study deserve to be optimized. Bonding vibrations between atoms constituting other polysaccharides, such as florid starch or cellulose, can interfere in the PCA and explain this distribution. Studies on the characterization of polysaccharides extracted from *S. chordalis* and purified over the seasons must be undertaken to confirm the presence of hybrid carrageenans and/or precursors during the “slow growth” of algae which begins at the end of autumn/winter [65].

### 3.3. Sargassum muticum 

*Sargassum* sp. contain alginate, laminarin(an), mannitol and fucoidan or other sulfated polysaccharides [66]. Mazumber et al. obtained an optimum alginate extraction yield of 13.57% from *S. muticum* harvested from the Nykøbing Mors coastal area in Denmark in the summer of August [67]. A similar result was obtained for *S. muticum* harvested in Spain in July using an alkaline extraction (13.6%) [68]. Nevertheless, the alginate yield of this species can show significant temporal fluctuations, from 11.14% in summer to 20.44% in winter according to a study on *S. muticum* from Morocco [69].

With regard to fucoidan extraction, yields ranged from 7.3% to 13.5% from *S. muticum* collected in Spain in August. The differences in yield can be explained by the extraction conditions, i.e., different extraction times and temperatures [70]. A similar result was obtained using ultrasound-assisted extraction for *S. muticum* collected in Spain in July (14.76%) [71].

We then compared our extracts with commercial standards of fucoidans from *Fucus vesiculosus*. The spectra are very similar, showing that the fucoidans of the different species of brown algae have a similar molecular structure. Moreover, these results confirm the quality of our extraction method and the purity of the samples obtained. Indeed, the main characteristic bands and absorbance spectra are correlated at about 70–80% between our extracts and the purified standards.

The same protocol was followed to compare the alginates extracted from *Sargassum* with a sodium alginate standard. This time, the correlation of the spectra exceeds 90%. We can therefore affirm that the FTIR technique has sufficient precision for the analysis of polysaccharides. This allowed us to make a detailed comparison of *Sargassum* fucoidans and alginates according to harvest season. We first noticed that all the fucoidans samples keep the same representative spectra as those observed with the standard (% correlation around 70%). However, there are slight variations in the appearance of the spectra depending on the month of harvest. These differences were plotted on a PCA graph which reveals a grouping into four classes corresponding to the four seasons of the year. Concerning the alginates, the variations according to the months of harvest highlighted by PCA are also very marked. These results indicate that the structure of polysaccharides changes with the season. Consequently, a modification of the biological activity of the molecules could be observed. This is not surprising because we had already observed such seasonal variations on the overall biochemical composition of *Sargassum* seaweed or on its protein composition [72]. However, these results on polysaccharides should be confirmed by the compilation of several successive years.

## 4. Materials and Methods

### 4.1. Materials

#### 4.1.1. *Eucheuma denticulatum*

*Eucheuma denticulatum* algae are harvested on the east coast of Madagascar, Sainte Marie Island, Analanjirofo region, Toamasina province, at geographic coordinates 16°59’37.28″ S, 49°53’5.13″ E, during the year 2021–2022, and they are harvested monthly. The algae harvested are wild algae from the reef near the Ilampy cultivation site in Ambodifotatra. The algae are then washed in fresh water to remove salts and other debris, and traditionally sun-dried for 4 days. The dried samples (around 500 g each) are then crushed and placed in vials with corresponding labels (scientific name, date, place of collection and other information about the algae) as follows: for the year 2021; (12 samples were collected): Jan 21, Feb 21, … Dec 21 and for the year 2022 (also 12 samples), Jan 22, Feb 22, …, Dec 22, so in total 24 samples were recorded and stored. Finally, they were freighted to the LBCM in Vannes, France, where they were freeze-dried in the laboratory and kept in the dark for later analysis.

#### 4.1.2. *Solieria chordalis*

Samples of *S. chordalis* were collected at the end of each month from January 2014 to October 2015. The collections took place at the same location at low tide in Saint-Gildas-de-Rhuys, Morbihan, Brittany (France), whose GPS coordinates are latitude 47°29’30.5″ N and longitude 2°49’52.0″ W. Between 2 and 5 kg of seaweed were harvested monthly. Then they were washed with tap water the same day and then subjected to the removal of epiphytes and sediment. Once washed, the seaweed was drained and air dried (18–22 °C) to remove any excess wash water. The dried algae were ground using a chopper (JUPITER T8) into small particles (diameter 3 mm) before freeze-drying (Alpha 1–4 −55 °C CHRIST). The dry powder is stored in the dark at room temperature (18–22 °C).

#### 4.1.3. *Sargassum muticum*

Sampling of *Sargassum muticum* (Yendo) Fensholt was carried out in Saint-Gildas-de-Rhuys (France) (47°30′03″ N, 2°50′12″ W) for one year (2015) in February, April, June and October.

The washed algae, with fresh water, were ground to a particle size of 3.0 mm. The grind was stored at −20 °C and then at −80 °C before freeze-drying. The freeze-dried algae were stored in a plastic bag away from direct sunlight.

### 4.2. Polysaccharide Extraction

#### 4.2.1. Carrageenans

Carrageenans from *E. denticulatum* were extracted using the standard classical hot extraction method [46,47] slightly modified. Briefly, dry algae (100 g) were depigmented with a 300 mL solution of acetone ≤ 99.8% (Fisher Scientific (Waltham, MA, USA), cas: 67-64-1) for 4 h in a Soxhlet mount and then the same method in absolute ethanol ≤ 99% (Fisher Scientific (Waltham, MA, USA), cas: 64-17-5). The depigmented algae were then extracted in 1.5 L of distilled water at 80 °C in a Thermomix for 4 h. The resulting solutions were then filtered through a cloth and the algae residues were re-extracted with distilled water in a Thermomix for 4 h at 80 °C. The filtrates obtained were separately precipitated overnight (in a cold room at 4 °C) with 99% absolute ethanol and stirred gently with a magnetic stirrer (kept overnight at 4 °C, before precipitation). The volume of ethanol was twice the volume of the filtrate and 4 teaspoons of NaCl were added. We then filtered the solution and the resulting solids were frozen at −80 °C for 1 h, freeze-dried, ground and stored in a dry place.

Concerning the polysaccharides extracted from *S. chordalis*, 5 g of ground and freeze-dried seaweed sample are inserted into a Soxhlet cartridge, which is then placed inside the Soxhlet extractor. An extraction of 1 h is carried out with 100% acetone. Then, a second extraction with a Soxhlet extractor is carried out for one hour with 150 mL of absolute ethanol. Residues depleted in pigments, lipids and proteins are obtained. These dried solid residues are immersed in 125 mL of distilled water and extracted at 80 °C for 2 h with continuous stirring. After filtration through gauze, the filtrate containing the polysaccharides is collected and cooled. A volume of absolute ethanol equal to twice the volume of the filtrate recovered is gradually added to the filtrate. Under strong manual stirring, the polysaccharides precipitate and form a ball around the glass stirrer. The precipitate is disaggregated and washed with ethanol. The resulting fibers are placed in an oven at 60 °C for one day. They are then finely ground in a mortar and stored away from light in a dry place at room temperature (18–22 °C) until use. For the same sample, the polysaccharide extractions were carried out in triplicate and the mass yields were expressed as a percentage of algal dry matter. The polysaccharide powders were analyzed by infrared spectrometer and then analyzed using the Unscrambler software. Polysaccharide yields and IR spectra were compared between samples harvested monthly (Jan = January; Feb = February; Mar = March; Apr= April; May; Jun = June; Jul =July, Aug = August; Sep = September; Oct = October; Nov = November; Dec = December).

#### 4.2.2. Alginates and Fucoidans

The parietal polysaccharides are extracted from 5 g of freeze-dried algae. They are first introduced into HCl (0.01 N, 75 mL) with 2% of CaCl_2_. After 2 h of extraction at room temperature with stirring, the solution is filtered and centrifuged (500 rpm, 5 min). The supernatant is collected and constitutes the soluble fucoidan fraction. This step is repeated with the remaining fraction, once at room temperature followed by two times at 70 °C. The residue constitutes the residual fraction and it is used for alginate extraction with Na_2_CO_3_ (3%, 75 mL) for two hours at 50 °C. This step is repeated four times and the soluble fraction collected constitutes the soluble alginate fraction.

All the soluble fractions are placed in a vacuum rotating flask for evaporation of the solvent. The fraction is dialyzed for four days in a dark and cold room in distilled water renewed daily. Finally, the dialysates were recovered and frozen for 48 h (−20 °C) before freeze-drying.

Analysis of sulfate compounds was performed by using the method of Jaques et al. (1968) [73] adapted by Pierre (2015) [61] and Burlot (2016) [54]. The aim of this assay is to determine the quantity of sulfate groups using a colorimetric method. In the aqueous phase, 3-amino-7-(dimethylamino) phenothizin-5-ium chloride (Azure A) will complex any sulfates that may be present, particularly within polysaccharides. The medium will develop a pink-violet color absorbing at λ = 535 nm, due to the formation of a chromophore in the presence of sulfates. The assay is semi-quantitative and gives an order of magnitude (~mg) of the sulfate concentration of a sample. Starting with a 1 mg/mL solution of sulfated dextran 17% (Sigma-Aldrich, St-Louis, MO, USA), a standard range is produced (0, 10, 20, 30, 40 and 50 µg/mL). In each well of the microplate, 20 µL of sample (1 mg/mL of aqueous hydrolysates) or standard and 200 µL of Azure A 10 mg/L aqueous solution are deposited. Optical density is measured against reagent blank at 535 nm using a microplate reader (Multiskan 60, Thermo Scientific, Waltham, MA, USA).

The amount of total sugars and related molecules in the samples was determined by the Dubois method [74], the principle of which is to transform sugar degradation products by the action of a strong acid into furfural derivatives. These chromogenic products are then condensed with phenol to obtain a chromophore. A range of glucose levels (0, 20, 40, 60, 80, 100 µg/mL) is used. Samples are acid hydrolysates prepared at 1 mg/mL. In a hemolysis tube, 1 mL of sample (previously diluted to the 10th) or standard is introduced. Then, 50 µL of phenol 75% (prepared from phenol crystals) and 2.5 mL of sulfuric acid (96%, d = 1.83) are added. The resulting solution is homogenized before being placed in an ice-water bath (to cool the tubes) for 10 min. The solution was then placed in a water bath at 30 °C for 10 min. Absorbance is measured at 490 nm against reagent white using a spectrophotometer (Spectro UV-1800, Shimadzu, Kyoto, Japan).

### 4.3. FTIR Analysis

FTIR spectra were recorded on a Nicolet iS5 FTIR spectrometer (Thermo Scientific, Waltham, MA, USA) using an iD7 ATR module with a diamond crystal. For the analysis of polysaccharides from *Eucheuma denticulatum* and *Solieria chordalis*, the dried powders were measured and compared to a standard ι- and κ-carrageenan standard (Sigma commercial grade). Alginates and fucoidans from *S. muticum* were also compared to standards. All spectra resulted from 16 scans and a resolution of 4 (0.482 cm^−1^). OMNIC 9.2.86 software (Thermo Scientific, Waltham, MA, USA) was used for data acquisition (absorbance mode). A background reference is obtained before each sample measurement. All the samples are recorded in triplicates.

### 4.4. Statistical Analysis

One-way analysis of variance (ANOVA) was used to find differences in carrageenan yield (SigmaPlot 12.0, Systat, Chicago, IL, USA). Significant differences between the different extracts were indicated by superscript letters at a significance level α = 0.05. The comparison includes the analysis of samples with n = 3 replicates.

Unscrambler X 10.4 software (Aspentech, Bedford, MA, USA) was used to synthesize the spectra using the PCA method.

## 5. Conclusions

Fourier Transform Infrared Spectrometry is an innovative technique for the determination of the biochemical composition of *Eucheuma denticulatum*, *Solieria chordalis* and *Sargassum muticum*, and in particular for the determination of the main characteristic absorption bands of polysaccharide compounds. Using this technique, we highlighted the variations in carrageenan, alginate and fucoidan composition of the three species of algae depending on the season. The advantages of FTIR spectroscopy are both speed and accuracy, as well as ease of use, and it can also be a useful tool for determining compositional variations in macroalgae. Therefore, IR spectroscopy can be used in the food, pharmaceutical and cosmetic industries to check the quality of phycocolloids by a non-destructive method. At present, FTIR spectroscopy is a qualitative measurement, but in the future, the prospects should be directed towards the quantitative assessment of polysaccharide compounds by regression methods using the specific absorption bands of the compounds studied.

## Figures and Tables

**Table 1:** Min and max values (% dry matter) measured for the biochemical composition of *Sargassum muticum* during one year.

Sulfate Compounds	Polysaccharides	Uronic Acids	Proteins	Mineral Matter	Total Nitrogen	Phenolic Compounds
6.9–9.6%	8.3–19.9%	1.5–4.1%	18.2–39.1%	18.8–28.9%	1.1–2.1%	2.3–5.1%

**Table 2:** Results of peak identification of infrared spectra for carrageenans (ι-,κ-,ν-) from *E. denticulatum* type ι-,κ-,ν- for the year 2021–2022.

Wavenumber (cm^−1^)	S=O (ι-,κ-,ν-)	DA* (ι-, κ-)	G/D (ι-,κ-,ν-)	DA (ι-, κ-)	DA2S*(ι-)	G/D6S* (ν-)	G4S (ι-,κ-,ν-)	G/D2S (ν-)	G/D6S (ν-)	DA2S (ι-)
IOTA Stand.	1218.05	1066.37	967.89	923.74	901.51	870.45	844.7	-	-	802.5
KAPA Stand.	1224.62	1063.61	970	921.27	-	874.26	843.27	-	-	-
Jan 21	1224.2	+	972.99	925.48	+	871.19	843.05	-	-	+
Jan 22	1221.5	1061.82	973.16	925.06	+	872.43	843	-	-	805.64
Feb 21	1224.38	+	973.79	925.47	+	+	841.56	-	-	+
Feb 22	1224.18	+	973.65	925.35	+	+	841.89	-	-	+
Mar 21	1223.62	+	972.63	926.13	+	+	841.39	-	-	+
Mar 22	1221.17	+	972.82	925.42	+	+	841.96	-	-	804.44
Apr 21	1224.21	+	973.61	926.02	894.52	871.33	842.61	-	-	+
Apr 22	1223.73	+	973.94	925.84	+	+	842.32	-	-	+
May 21	1218,36	1061	976.18	924.64	891.69	+	841.77	-	-	804.49
May 22	1224.86	+	974.8	925.67	894.01	+	842.21	-	-	803.39
Jun 21	1224.9	+	973.93	925.74	894.93	+	842.84	-	-	+
Jun 22	1224.13	1062.04	972.98	924.82	892.62	+	843.37	-	-	805.7
Jul 21	1220.79	1059.01	973.29	925.09	894.19	+	842.52	-	-	803.31
Jul 22	1224.34	+	973.86	925.92	894.76	+	842.74	-	-	+
Aug 21	1222.91	1061.39	975.8	925.24	892.8	870.02	843.02	-	-	804.79
Aug 22	1224.05	1061.96	975.13	925.24	893.66	872.58	843.16	-	-	+
Sep 21	1224.39	1066.37	974.38	925.98	+	+	842.61	-	-	+
Sep 22	1221.76	+	974.28	925.68	895.14	+	842.63	-	-	+
Oct 21	1221.15	+	974.53	925.24	894.36	+	842.28	-	-	805.41
Oct 22	1222.94	1062.19	973.39	925.14	893.76	871.16	842.89	-	-	805.87
Nov 21	1224.07	+	973.29	926.15	+	871.7	842.23	-	-	+
Nov 22	1223.45	+	973.57	926.13	895.96	+	842.1	-	-	+
Dec 21	1224.26	+	974.01	926.15	+	873.21	843.06	-	-	+
Dec 22	1224.48	+	973.22	925.62	894.71	870.26	843.14	-	-	+

* Shouldered; -: Absent peak; +: Presence of peak (low intensity).

**Table 3:** Percentages of similarity of samples (triplicates) with standard fucoidans.

February	April	June	October
72.74%	72.35%	64.41%	75.60%
72.66%	73.13%	68.48%	75.70%
71.54%	73.21%	67.06%	75.99%
